# Phylogenomic Comparison of Seven African Swine Fever Genotype II Outbreak Viruses (1998–2019) Reveals the Likely African Origin of Georgia 2007/1

**DOI:** 10.3390/pathogens12091129

**Published:** 2023-09-04

**Authors:** Rivalani F. Mthombeni, Armanda D. Bastos, Antoinette van Schalkwyk, Juanita van Emmenes, Livio Heath

**Affiliations:** 1 Agricultural Research Council—Onderstepoort Veterinary Institute, Onderstepoort 0110, South Africa; mthombenir@arc.agric.za (R.F.M.); vanschalkwyka1@arc.agric.za (A.v.S.); vanheerdenj@arc.agric.za (J.v.E.); 2 Department of Zoology & Entomology, Faculty of Natural and Agricultural Sciences, University of Pretoria, Pretoria 0002, South Africa; 3 Department of Veterinary Tropical Diseases, Faculty of Veterinary Science, University of Pretoria, Pretoria 0110, South Africa; 4 Department of Biotechnology, University of the Western Cape, Bellville 7535, South Africa

**Keywords:** African swine fever virus, genotype II, complete genome sequencing, phylogenetics, single nucleotide polymorphisms (SNPs)

## Abstract

Since the initial report of African swine fever (ASF) in Kenya in 1921, the disease has predominantly been confined to Africa. However, in 2007, an ASF genotype II virus of unknown provenance was introduced to Georgia. This was followed by its rampant spread to 73 countries, and the disease is now a global threat to pig production, with limited effective treatment and vaccine options. Here, we investigate the origin of Georgia 2007/1 through genome sequencing of three viruses from outbreaks that predated the genotype II introduction to the Caucasus, namely Madagascar (MAD/01/1998), Mozambique (MOZ/01/2005), and Mauritius (MAU/01/2007). In addition, genome sequences were generated for viruses from East African countries historically affected by genotype II (Malawi (MAL/04/2011) and Tanzania (TAN/01/2011)) and newly invaded southern African countries (Zimbabwe (ZIM/2015) and South Africa (RSA/08/2019). Phylogenomic analyses revealed that MOZ/01/2005, MAL/04/2011, ZIM/2015 and RSA/08/2019 share a recent common ancestor with Georgia 2007/1 and that none contain the large (~550 bp) deletion in the *MGT110 4L* ORF observed in the MAD/01/1998, MAU/01/2007 and TAN/01/2011 isolates. Furthermore, MOZ/01/2005 and Georgia 2007/1 only differ by a single synonymous SNP in the *EP402R* ORF, confirming that the closest link to Georgia 2007/1 is a virus that was circulating in Mozambique in 2005.

## 1. Introduction

African swine fever (ASF), caused by African swine fever virus (ASFV), is a haemorrhagic fever of domestic pigs with mortality rates approaching 100% [1,2]. This unique virus belongs to the monotypic genus *Asfivirus* within the *Asfarviridae* family, and it is currently the only known double-stranded deoxyribonucleic acid (dsDNA) arthropod-borne virus (arbovirus) [2,3]. The ASFV genome ranges from 170 to 193 kilobase pairs (kbp) in size, encodes more than 180 putative open reading frames (ORFs) and replicates in the cytoplasm of infected cells [3,4,5]. The genome has a conserved central region and two variable ends referred to as the left and right variable regions [2,6].

ASFVs group within 24 genotypes (I–XXIV) based on the partial sequences of the C-terminal region of the B646L (*p72*) gene encoding virus protein 72 (VP72) [7,8,9,10,11]. Of the 24 known genotypes, genotype II is the most widely distributed and displays high levels of sequence identity across the gene markers most commonly used for genotype assignment and within-genotype resolution [12,13]. The high levels of homogeneity and limited number of genotype II viruses of African origin predating the 2007 excursion to the Caucasus have impeded identification of the most likely origin of the virus introduced to Georgia in 2007. Despite this, the availability of B646L/*p72* and central variable region (CVR)/*B602L* data from Mozambique and other East African countries, and for outbreak strains from Madagascar provided the first indication that the origin of Georgia 2007/1 was from the eastern seaboard of Africa [13,14]. A subsequent study in Mozambique revealed that it was not possible to clearly differentiate closely-related genotype II viruses with the two genetic markers, *p72* and CVR, that are used for genotype assignment and intra-genotypic resolution, respectively [11]. However, partial sequencing of the B646L/*p72* region of genotype II viruses from diverse African countries confirmed that these ASFVs are homogeneous and share common ancestry [11,13,14,15].

The first report of ASFV genotype II in Georgia in 2007 marked the start of a major incursion into Eastern Europe, Russia, and later Asia, Western Europe and the Dominican Republic [14,16,17,18,19,20]. Due to the devastating impact of ASF in Eurasia, intensive efforts have been directed towards generating complete genome sequences to reconstruct the evolutionary relatedness of viral isolates responsible for outbreaks in affected countries. These efforts have been met with limited success in tracing the source and transmission pathways of the introduced virus due to the high levels of genome homogeneity [5,18,19]). Currently, the majority of available full genome sequences for genotype II ASF viruses are of Eurasian origin, and data for viruses of African origin are limited to five representatives from Tanzania, and Malawi, viz. Tanzania/Rukwa/17/1, TAN/17/Mbagala, TAN/17/Kibaha, TAN/20/Mogorogo, and MAL/19/Karonga, and one from Nigeria, RV502 (OP672342) [21,22,23,24]. These five genotype II viruses of East African origin are distinct from Georgia 2007/1, based primarily on differences in *K177R* gene sequence [22,23]. Differences between the virus genome sequences from Tanzania and Georgia 2007/1 arise from one large insertion/deletion (indel) and various single nucleotide polymorphisms (SNPs). Thus, it is clear that despite the large number of available genotype II genome sequences from Europe and Asia, the paucity of data from Africa has meant that insight into the evolutionary relationships between ASFV isolates from south-eastern Africa and the geographical origin of the virus introduced into Georgia in 2007, remain unknown.

The aim of this study was to investigate the origin of Georgia 2007/1 and the evolutionary relationships of south-east African outbreak strains by sequencing ASFVs from seven southern African development community (SADC) countries (https://www.sadc.int/member-states (accessed on 31 July 2023)) affected by genotype II. This was achieved by generating complete ASFV genome sequences for three viruses from Mozambique, Madagascar and Mauritius, which predate the 2007 excursion from Africa to Georgia, and four additional viruses (2011–2019) from the two SADC countries historically affected by genotype II (Malawi and Tanzania; 2011) and the two newly-affected countries (Zimbabwe and South Africa; 2015 and 2019, respectively).

## 2. Material and Methods

### 2.1. Sample Collection

Seven ASFV isolates were selected from the virus repository at the World Organisation for Animal Health (WOAH) Reference Laboratory for ASF at the Transboundary Animal Diseases Laboratory of the Agricultural Research Council—Onderstepoort Veterinary Research (ARC-OVR) in South Africa. These isolates were selected based on temporal and geographical distribution to ensure the representation of seven of the eight SADC countries in which genotype II outbreaks have been reported/recorded. The viruses span a 21-year period (1998–2019; Table 1).

### 2.2. Sample Preparation for NGS

The viruses were cultured in primary bone marrow cells (PBMCs), harvested, and purified using a sucrose gradient purification method, as previously described [25,26]. Viral DNA was extracted using the high pure PCR template preparation kit (Roche, Basel, Switzerland) according to manufacturer’s instructions, and the concentration was determined using a NanoDrop^®^ ND-1000 UV-Vis Spectrophotometer. As the DNA concentration was not sufficient for downstream NGS, whole genome amplification was performed using the Illustra Genomiphi V3 DNA amplification kit (GE-Healthcare, Thermo fisher Scientific, Waltham, MA, USA) using prescribed reaction conditions.

### 2.3. NGS and Data Analysis

The viral DNA was used to construct a sequencing library using the Truseq Nano DNA library preparation kit (Illumina, San Diego, CA, USA) according to manufacturers’ instructions. The libraries were quality controlled using the Qubit fluorometer (Invitrogen, Waltham, MA, USA) and PerkinElmer LabChip (PerkinElmer, Singapore) instruments. Sequencing was performed using an Illumina HiSeq 2500 instrument (Illumina, San Diego, CA, USA) using the V4 SBS chemistry, generating 2× 125-bp paired-end reads for each of the samples. Data generated for all seven viruses were analysed using CLC Genomics workbench v9.5.2 (CLC bio, Qiagen, www.clcbio.com (accessed on 31 July 2023)). Between 41,566,834 and 131,025,188 reads were obtained for each of the samples. Low quality reads and adaptor sequences were removed prior to mapping against the reference sequence Georgia 2007/1 (FR682468.2) and extracting the consensus sequence. De novo assembly was also performed with the reads, and the resulting consensus sequence was compared to the one generated from the mapping analysis. Deletions, insertions and unique point mutations were verified through PCR-Sanger sequencing, with the major deletion described in Section 2.5 below, prior to generating a final, single consensus sequence for each virus. The trimmed reads were then re-mapped to the final sequence in order to determine the average coverage of the reads. Open reading frames were predicted using Georgia 2007/1 (FR682468.2) as the reference, and the complete genome sequences were submitted to GenBank under the accession numbers indicated in Table 1.

### 2.4. Phylogenetic Analyses

Complete genome sequences from genotype II ASFs representing Africa, Georgia 2007/1 (FR682468.2) and representative genomes of the three geographical clades previously described in Europe, Russia and Asia were obtained from GenBank and used to create an alignment with the newly generated consensus sequences (Supplementary Table S1). The alignment was used to identify novel SNPs and indels, and for phylogenetic analyses. Phylogenomic trees were inferred using maximum likelihood, and bootstrap support was estimated through 100 non-parametric iterations under the General Time Reversible (GTR, G + I = 4) model in Mega X [27]. In addition, individual gene phylogenies were inferred for the traditional gene targets (*p72*, *p54*, CVR and *p30*) and for newly advocated gene targets (ORFs consist of EP402R, MGF-360-13L, A859L, O61R, MGF-360-9L, G1211R and I267L as well as the IGR between ORFs I73R and I329L).

### 2.5. Confirmation of a 550 bp Deletion Using PCR-Sanger Sequencing

An approximately 550 bp deletion was detected in MAD/01/1998, MAU/01/2007 and TAN/01/2011 in relation to Georgia 2007/1. In order to validate the deletion, primers were designed to bind to regions flanking the deletion. Primers ASFV6980-F: 5′-CAT ACA GTG TTC CAT GGG ATA-3′ and ASFV8820-R: 5′-GGA CAA CTT CAT CCA ACG G- 3′ were each used at 0.5 µM, with 2 µL DNA template in a 25 μL reaction containing 12.5 μL GoTaq green master mix (Promega, Singapore). Amplification was performed using a T100 Thermal cycler (Bio-Rad, Hercules, CA, USA) at an annealing temperature of 55 °C. The PCR amplicons were run against the O’GeneRuler DNA Ladder Mix (Thermo Fisher Scientific, Waltham, MA, USA) and visualised on a 1% agarose gel stained with ethidium bromide prior to submission to Inqaba Biotech, Pretoria, South Africa (https://inqababiotec.co.za/ accessed on 31 July 2023) for Sanger sequencing.

## 3. Results

### 3.1. Phylogenomic Comparison of ASFVs

The selected viruses were passaged on PBMCs, genomic DNA was extracted and NGS were performed on each of the isolates in order to generate a single consensus sequence represented by an average coverage of more than 36.1 reads. The annotated sequences were submitted to GenBank (Table 1) and used in subsequent phylogenetic analysis along with sequences representing genotype II ASFV from Africa, Europe and Asia (Supplementary Table S1).

**Figure 1:**
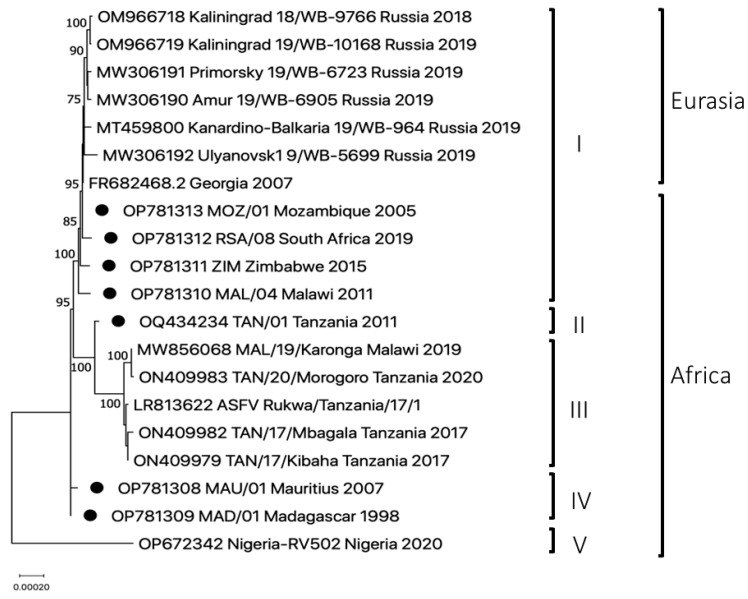
Maximum likelihood phylogenetic tree using the complete genome sequences of genotype II ASFVs from seven SADC countries, Georgia, Russia and Nigeria. The complete genome sequences generated in this study are indicated with black dots, whilst the remaining sequences were obtained from GenBank. Bootstrap support values from 100 replications, ≥75 are shown next to the relevant nodes, with the five major clades recovered being indicated by Roman numerals (I–V).

Phylogenomic analyses recovered five discrete clades (I–V; Figure 1). There was a clear phylogenetic separation between previously published virus genome sequences from recent outbreak strains (2017–2020) from Tanzania and Malawi (LR813622, ON409982, ON409983, ON409979 and MW856068), and the seven newly generated sequences from the SADC region. Six of the seven genome sequences clustered with Georgia/2007 and other genotype II viruses from Europe and Asia within clade I. In contrast, TAN/01/2011 formed a monophyletic lineage distinct from the two previously described major clusters and was assigned to clade II (Figure 1). Interestingly, MAD/01/1998 and Mau/01/2007 clustered together distinct from the viruses from the SADC region (clade IV), suggesting that the ASFVs responsible for outbreaks of the disease on these islands were genetically distinct from the genotype II viruses circulating at around the same time on mainland Africa.

**Figure 2:**
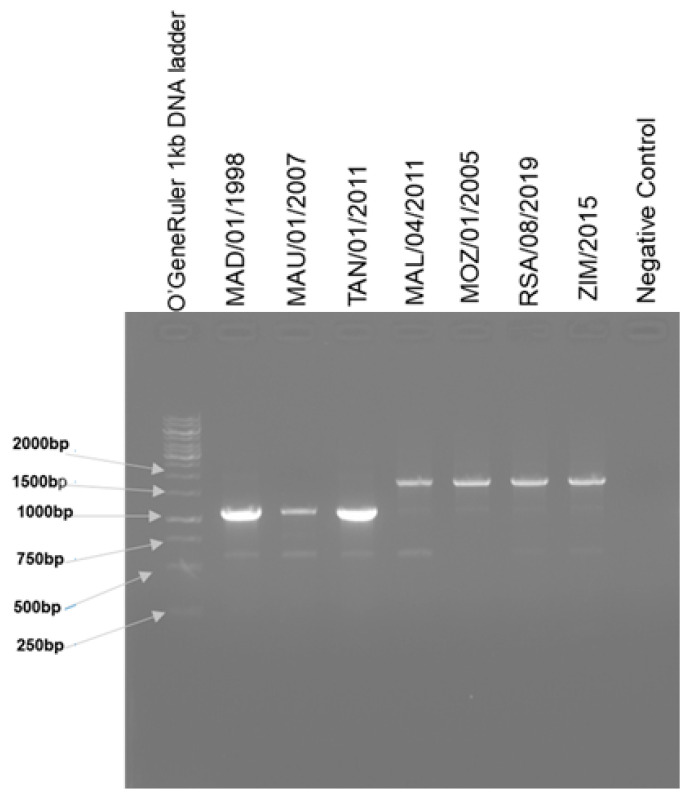
Agarose gel indicating the size difference pertaining to the approximately 550 bp deletion in MAD/01/1998, MAU/01/2007 and TAN/01/2011 when compared to MOZ/01/2005, MAL/04/2011, RSA/08/2019 and ZIM/2015. A no-template negative control was included.

The 5′ terminal region of sequences MAU/01/2007, MAD/01/1998 and TAN/01/2011 had an approximately 550 bp deletion in the MGF 110-4L gene region that is identical to a deletion previously described in genotype II genome sequences from East Africa, (Tanzania/Rukwa/17/1, TAN/17/Mbagala, TAN/17/Kibaha, TAN/20/Mogorogo, and MAL/19/Karonga). Amplification using primers flanking the deleted region, resulted in shorter amplicons for MAU/01/2007, MAD/01/1998, and TAN/01/2011, compared to the other four viruses, thus confirming the ~550 bp deletion (Figure 2).

### 3.2. Individual Gene Phylogenies and SNP Detection

**Figure 3:**
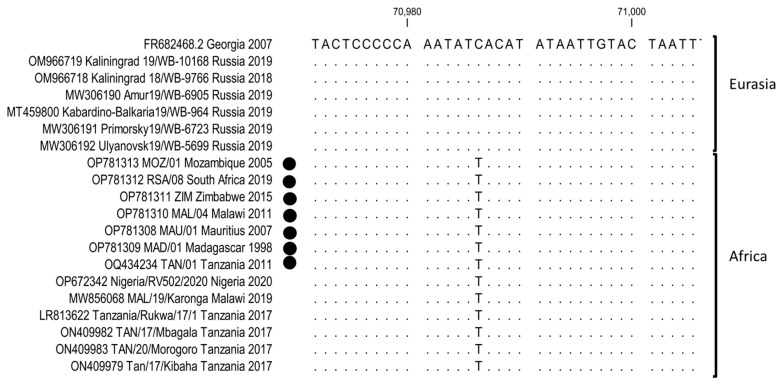
Sequence alignment of ORF *EP402R* from ASFVs isolated in Africa and Eurasia. The genome sequences generated in this study are denoted by a closed circle.

The newly-generated complete genome sequences were compared against the available genotype II ASFVs sequences, with special emphasis to the SNPs between Georgia 2007/1 (FR682468.2) and the African isolates. A single synonymous SNP in the open reading frame (ORF) *EP402R* (*I118I*) was identified between MOZ/01/2005 and Georgia 2007/1 (Supplementary Table S2). This SNP was a “T” in all the isolates from Africa, compared to a “C” in Georgia 2007/1 as well as all the isolates from Eurasia (Figure 3). Additionally, 51 non-synonymous (Supplementary Table S3), 40 synonymous (Supplementary Table S2) and 6 single nucleotide polymorphisms (SNPs) altering reading frames, and 18 SNPs in intergenic regions (Supplementary Table S4), were observed. SNPs were identified between the newly generated sequences and Georgia 2007/1. The sequence ZIM/2015 had 11 unique non-synonymous and three unique synonymous SNPs (Supplementary Tables S2 and S3). In contrast, RSA/08/2019 had seven unique non-synonymous and five unique synonymous SNPs, whilst MAL/04/2011 had nine and six, respectively. No unique SNPs were identified in MAD/01/1998, but all the SNPs detected in this sequence were also observed in MAU/01/2007 (Supplementary Tables S2 and S3). Four synonymous SNPs were unique to TAN/01/2011, whilst it shared 15 non-synonymous and ten synonymous SNPs with the previously determined sequences from Tanzania and Malawi (LR813622, ON409982, ON409983, ON409979 and MW856068) (Tables S2 and S3).

**Figure 4:**
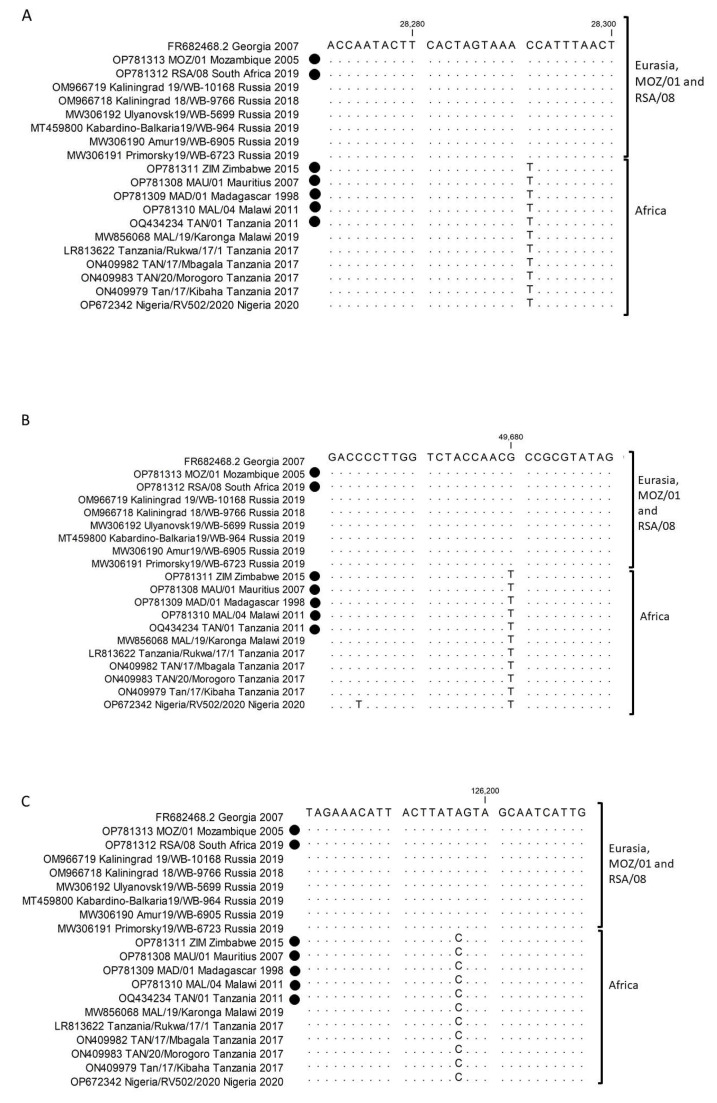
Sequence alignment of ORFs MGF-360-13L (**A**), A859L (**B**) and O61R (**C**). The sequences of MOZ/01/2005 and RSA/08/2019 cluster with Georgia 2007/1 and the selected Eurasian isolates, whilst the remainder of African isolates cluster together. The genome sequences generated in this study are denoted by a closed circle.

**Figure 5:**
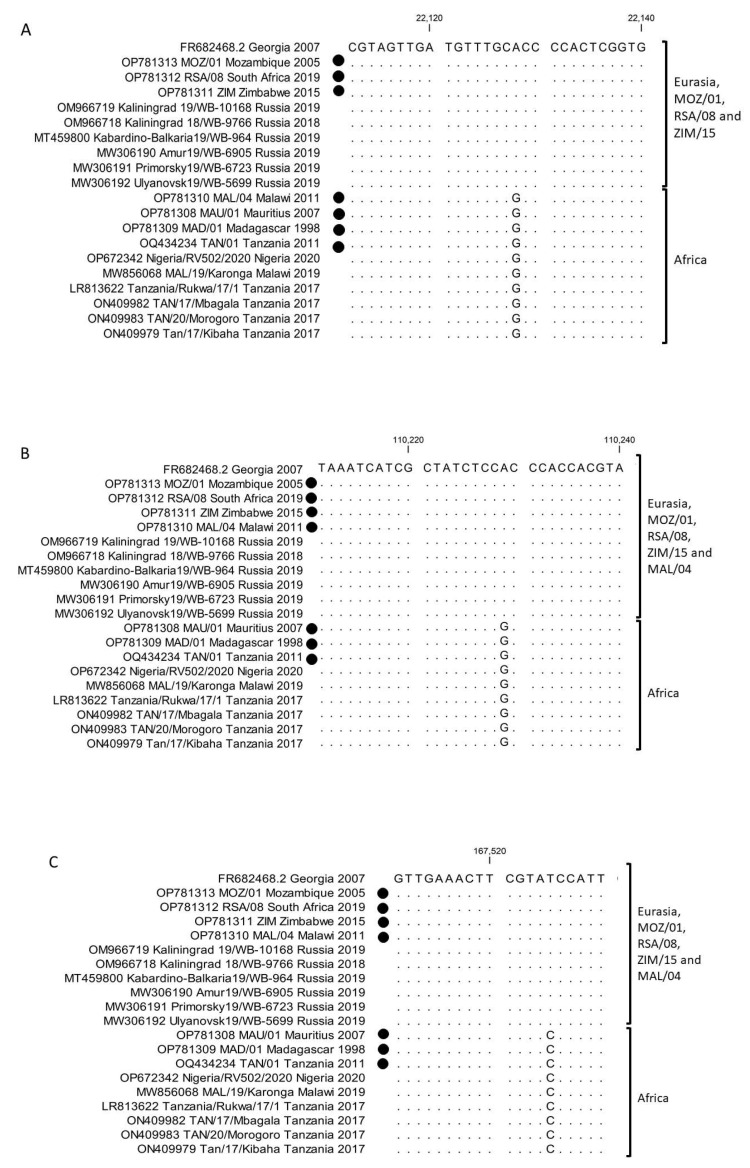
Sequence alignment of ORFs MGF-360-9L (**A**), G1211R (**B**) and I267L (**C**). The sequences of MOZ/01/2005, RSA/08/2019 and ZIM/2015 cluster with Georgia 2007/1 (**A**), whilst the sequences of MOZ/01, RSA/08, ZIM/2015 and MAL/04 cluster with Georgia 2007 and the selected Eurasian isolates (**B**,**C**). In contrast, the remainder of African isolates cluster together. The genome sequences generated in this study are denoted by a closed circle.

The polymorphisms were subsequently evaluated for their potential role as markers to delineate the isolates from Africa. Three of the SNPs (MGF360-13L: G177D), (A859L: A427E) and (O61R: I181I) were discriminated between a group consisting of Georgia 2007/1, MOZ/01/2005 and RSA/08/2019 compared to the remaining sequences from Africa (Supplementary Table S2; Figure 4). Additionally, three SNPs, (MGF-360-9L: G79G), (G1211R: N1002I) and (I267L: I129V) for ZIM/2015, and two SNPs (G1211R: N1002I) and (I267L: I129V) (Supplementary Table S2; Figure 5) for MAL/04/2011 clustered them into the Georgia 2007/1 group.

**Figure 6:**
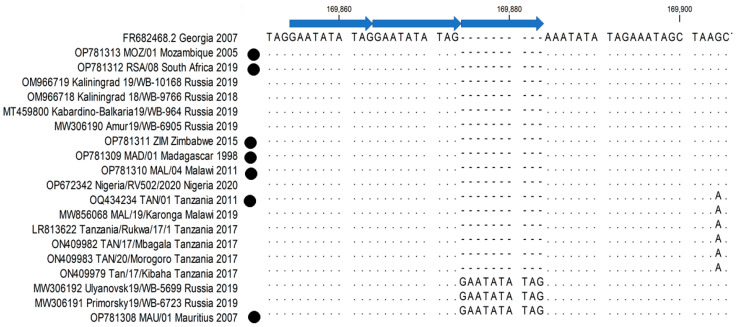
Sequence alignment of the IGR between ORFs I73R and I329L, with the 10 bp repeat regions indicated with blue arrows. The genome sequences generated in this study are denoted by a closed circleFifteen of the 51 non-synonymous SNPs observed in the sequences under study were non-conservative replacements. The results from this study showed that within the MGF100-3L, MGF110-3L and MGF110-5L-6L, ZIM/2015 had amino acid substitutions as aspartic acid (D) at position 85 replacing glycine (G) (D85G), G91D and leucine (L) at position 35 replacing histidine (H) (L35H), respectively, whereas in MGF360-13L, the substitution of G177D occurred in all the African isolates except for MOZ/01/2005 and RSA/08/2019, which were the same as Georgia 2007/1. Similarly, alanine (A) at position 427 replaced glutamic acid (E) (A427E) within the ORF A859L, occurring in the same manner as mentioned above. Other non-synonymous SNPS, where non-conservative replacement occurred, involved isolate MAL/04/2011, which is where Q115R, R39Q, aspartic acid (D) at position 35 replaced asparagine (N) (D35N) and R42G, occurring in MGF110-13LB, MGF505-9R, MGF360-2L and MGF360-19Rb, respectively. In RSA/08/2019, D151N in the ORF B169L, and E125V in E199L; and in ZIM/2015, L351D in the ORF QP509L and Q104H in E199L, non-conservative replacements occurred. TAN/01/2011 had an amino acid exchange of W109R in MGF 110-1L, the absence of serine in position 164 of MGF 110-5L-6L, threonine (T) at position 81 replaced by methionine (M) [T81M] in MGF110-8L, G177D in MGF 360-13L, T78M in C275L, N1002I in G1211R, M288T in D245L, D130N in E165R, and A83T in MGF505-11L.

An analysis of the intergenic regions (IGRs) indicated two SNPs and two indels in the sequence of TAN/01/2011, which cluster this isolate with previously determined sequences from Tanzania and Malawi (LR813622, ON409982, ON409983, ON409979 and MW856068) (Supplementary Table S4). Interestingly, MAU/01/2007 had three copies of the (GAATATATAG) 10 bp tandem repeat sequences (TRS) in the IGR between ORFs I73R and I329L, characterising this isolate as IGR-II (Supplementary Table S4; Figure 6).

## 4. Discussion

Since the first introduction of genotype II ASFV to Georgia in 2007, the disease has spread across the Caucasus, Europe, Russia, Asia and the Dominican Republic [20,28]. The complete genome sequence analysis of ASFVs from different geographical locations and temporal distributions indicate that virus populations were accumulating mutations at regular intervals. These SNPs enabled the differentiation of the circulating Genotype II ASFVs into an Eastern, Western and most recently a far-Western cluster [29,30]. Despite this, the origin and common ancestor to ASFV introduced into Georgia has not been confirmed. In order to address this gap and expand our current understanding of ASFV evolution, this study determined the complete genome sequences of seven southern genotype II ASFVs from Africa. Based on the original epidemiological back tracing performed after the 2007 outbreak in Georgia [14], ASFVs from Mozambique in 2005 and Mauritius in 2007 were selected for sequencing. The first outbreak of ASF on the island republic of Mauritius followed a few months after an outbreak on the island of Madagascar [31]. Based on a combination of single marker analyses, it was suggested that the Madagascar/2007 and Mauritius/2007 outbreaks were epidemiologically related, resulting in the selection of an isolate from the first genotype II outbreak in Madagascar in 1998 rather than the 2007 outbreak [7,13,31]. This allowed for an enhanced spectrum of the genomic diversity of genotype II ASFVs circulating in the SADC region to be obtained. In addition to Mauritius/2007, two other genotype II ASFVs reported for the first time in Zimbabwe in 2015 [15] and South Africa in 2019, were included in this study. Recently, sequences of ASF genotype II viruses isolated in Tanzania and Malawi were published (LR813622, ON409982, ON409983, ON409979 and MW856068) [21,22,23]. Significant sequence differences were described between the African isolates and Georgia 2007, prompting this study to include a virus from Malawi and Tanzania to increase the temporal spectrum of viruses from that region.

The complete genome analyses performed in our study indicate that isolates from Mozambique (2005) and South Africa (2019) are more closely related to the Georgia 2007 virus than the latter is to the viruses currently circulating in Europe and Asia (Figure 1). The full genome sequences provided additional novel insights into the evolution of genotype II ASFVs in Africa and assisted with refining the provenance of the Georgia 2007 virus. Based on the data generated in this study, it is evident that the virus introduced into Georgia in 2007 was from southeast Africa. Georgia 2007/1 and MOZ/01/2005 only differ by one SNP, suggesting the latter was either the source or a close relative to ASFV initially introduced into Georgia. A comparison between Georgia 2007/1 and subsequent samples obtained in 2019 from both South Africa (this study) and Russia [29] indicated a higher percentage sequence similarity with RSA/08/2019 (99.99%) than any of the sequences from the Russian Federation (99.97–99.98%). This suggests a higher substitution rate in Europe and Asia compared to Africa, which could be related to an increase in virus population number in Eurasia or due to transmission via the sylvatic cycle in Africa [9,32].

The viruses from South Africa (RSA/08/2019), Zimbabwe (ZIM/2015) and Malawi (MAL/04/2011), shared a recent common ancestor with Georgia 2007/1 and Mozambique (MOZ/01/2005); the percentage sequence identity ranged from 99.68% to 99.99% amongst the five ASFVs. In contrast to these closely related ASFVs, the sequences of isolates MAD/01/1998, MAU/01/2007 and TAN/01/2011 shared a recent common ancestor with the previously published viruses from Tanzania and Malawi (Tanzania/Rukwa/17/1 (LR813622), MAL/19/Karonga (MW856068), TAN/17/Mbagala (ON409982), TAN/17/Kibaha (ON409979) and TAN/20/Mogorogo (ON409983). The divergence observed amongst the genotype II ASFVs from Africa further indicates that the Madagascar/2007 outbreak might have been related to the previous 1998 outbreak on the island, rather than a new introduction related to MOZ/01/2005 and subsequently Georgia 2007/1 ASFVs. A deletion of ~550 bp was detected in the genotype II genome sequences for Madagascar (MAD/01/1998), Mauritius (MAU/01/2007) and Tanzania (TAN/01/2011), which corresponds to a previously described deletion in the 5′ region of Tanzania/Rukwa/17/1, MAL/19/Karonga, TAN/17/Mbagala, TAN/17/Kibaha and TAN/20/Mogorogo [21,22,23].

Additionally, a significant difference between MAL/04/2011 (this study) and MAL/19/Karonga [21] was observed, despite both isolates originating from Malawi. As previously mentioned, MAL/04/2011 clustered with Georgia/2007, MOZ/01/2005, RSA/08/2019 and ZIM/2015 in contrast to MAL/19/Karonga, which clustered with MAD/01/1998, MAU/01/2007 and all the sequences from Tanzania. This suggests a probable new introduction of ASFV genotype II from southern Africa to Malawi within the eight-year period. This is based on the vast number of SNPs as well as the absence of the 650 bp deletion in the ORF MGF110-4L of MAL/04/2011 in comparison to MAL/19/Karonga. These results indicate the need for additional genotype II genome sequencing of viruses from multiple outbreaks in all the SADC countries.

A comparison of the newly sequenced ASFVs to Georgia 2007/1, indicated 18 SNPs in the IGRs (Supplementary Table S4), and 51 non-synonymous (Supplementary Table S2) and 40 synonymous SNPs (Supplementary Table S3). In contrast, previous sequences from isolates in the eastern and western regions of Russia had 13 SNPs in the IGR, and 22 non-synonymous and 15 synonymous SNPs [29]. This is despite the 21-year temporal distribution of the African samples (1998–2019) in comparison to the 12 years (2007–2019) that viruses were circulating in Eurasia [29]. Interestingly, non-synonymous SNPs were detected in the ORFs (MGF-505-9R, I267L and E199L) between both the Eurasian and African datasets [29]. The ORF (MGF-505-9R) previously had E323K and Y456H exchanges described, whilst this study described a R39Q exchange in MAL/04/2011 (Supplementary Table S2). Similarly, an I195F exchange in the ORF I267L has been described, whilst a I129V exchange separates the MAU/01/2007 and MAD/01/1998 samples from the remaining SADC samples and Georgia 2007/1 (Supplementary Table S2). The ORF E199L had a Q104H amino acid exchange described in both ASFV/Primorsky 19/WB-6723 and ASFV/KabardinoBalkaria 19/WB-964. This histidine exchange was as a result of either a (CAT) or (CAC) substitution, instead of the glutamine (CAA) [29]. ZIM/2015 had the same Q104H amino acid exchange, due to a histidine (CAT) substitution, similar to ASFV/Primorsky 19/WB-6723. The effect of this amino acid exchange on the possible antigenicity of the virus envelope protein has been discussed in detail previously [29].

Complete genome sequence analysis remains the gold standard in identifying novel SNPs and determining the evolution and molecular epidemiology of a virus population. Unfortunately, this is time-consuming and expensive, resulting in the study of individual markers to ascertain the spread and epidemiology of ASFVs during outbreaks. Various markers have been described to delineate the genotype II ASFVs circulating in Europe and Asia [28,30]. These genome markers focused on differences in the IGRs between I73R and I329L as well as between ORF MGF 505-9R and I267L. MAU/01/2007 clustered within group IGR-II, based on the previously described 10 bp indel in the IGR between I73R and I329L [32], whilst in the same IGR an A to G SNP separated TAN/01/2011 together with the other previously sequenced ASFVs from Africa from the rest of the sequences generated in this study. The ORF I267L was previously described as marker due to the I195F exchange in Eurasian isolates [28], whilst this study identified a unique *240 W in MAL/04/2011 as well as an I129V exchange separating the African isolates in two groups, one consisting of ZIM/2015, MOZ/01/2005, RSA/08/2019 and MAL/04/2011. These were the only markers previously described in Eurasia, capable of differentiating between sequences from Africa, suggesting that the previously described markers are unique in samples from outbreaks following the initial introduction into Georgia, 2007 [28].

In conclusion, this study provides novel epidemiological findings on the origin of the virus introduced into Georgia in 2007. Due to a single synonymous SNP in the ORF *EP402R* between MOZ/01/2005 and Georgia 2007/1, it is suggested that a possible link exists between the founder ASFV in Georgia 2007/1 and viruses circulating concurrently in Mozambique. The study proved that the Georgia 2007/1 virus shared common similarities with RSA/08/2019 (South Africa), ZIM/2015 (Zimbabwe), MAL/04/2011 (Malawi) and MOZ/01/2005 (Mozambique). With regard to the evolutionary relationship between Georgia 2007/1 and ASFVs from this region, the study found a major divergence in viruses before and after Georgia 2007/1, highlighting the presence of ASFV genotype II clusters in Africa. These results underscore the need for sequencing additional genotype II strains from Africa with particular focus on earlier isolates in order to determine how diverse this genotype is.

## Figures and Tables

**Table 1:** Summary of the ASFV genotype II isolates from SADC with the GenBank accession numbers of the complete genome sequences generated in this study.

Isolate Name	Country of Origin	Year Isolated	Host Species	*p72* Genotype	GenBank Accession Number
MAD/01/1998	Madagascar	1998	Domestic pig	II	OP781309
MOZ/01/2005	Mozambique	2005	Domestic pig	II	OP781313
MAU/01/2007	Mauritius	2007	Domestic pig	II	OP781308
MAL/04/2011	Malawi	2011	Domestic pig	II	OP781310
ZIM/2015	Zimbabwe	2015	Domestic pig	II	OP781311
RSA/08/2019	South Africa	2019	Domestic pig	II	OP781312
TAN/01/2011	Tanzania	2011	Domestic pig	II	OQ434234

## Data Availability

All virus genome sequences generated in this study were submitted to Genbank and are available under the accession numbers indicated in Table 1.

## Supplementary Materials

The following supporting information can be downloaded at: https://www.mdpi.com/article/10.3390/pathogens12091129/s1. Table S1: Genome sequences included in this study and their corresponding Genbank accession numbers; Table S2: Synonymous SNPs of seven ASFVs under study compared to Georgia 2007/1 (FR682468.2), Tanzania/Rukwa/17/1 (LR813622) and MAL/19/Karonga (MW856068) with nucleotide substitutions on amino acids highlighted in grey; Table S3: Non-synonymous SNPs identified between nine ASFV genotype IIs from Africa compared to Georgia 2007/1; Table S4: SNPs observed within the intergenic regions of the nine African Isolates and Georgia 2007/1 with differences highlighted in grey.
