# Supplementary material for: Phylogenomic Comparison of Seven African Swine Fever Genotype II Outbreak Viruses (1998–2019) Reveals the Likely African Origin of Georgia 2007/1
Source: Pathogens. 2023 Sep 4;12(9):1129. doi: 10.3390/pathogens12091129 (PMC10537866; doi:10.3390/pathogens12091129)
Supplement: Supplementary file 1 [file pathogens-12-01129-s001.zip › pathogens-2562372-supplementary.pdf]

pathogens

MDPI

Supplementary Table S1. Genome sequences included in this study and their correspodning GenBank accession numbers

Isolate Name	Country of Origin	Year Isolated	GenBank Accession Number
Georgia/2007	Georgia	2007	FR682468.2
Tanzania/Rukwa/17/1	Tanzania	2017	LR813622
Tan/17/Kibaha	Tanzania	2017	ON409979
TAN/17/Mbagala	Tanzania	2017	ON409982
TAN/20/Morogoro	Tanzania	2020	ON4009983
MAL/19/Karonga	Malawi	2019	MW856068
Nigeria/RV502/2020	Nigeria	2020	OP672342
Primorsky19/WB-6723	Russia	2019	MW306191
Ulyanovsk19/WB-5699	Russia	2019	MW306192
Amur19/WB-6905	Russia	2019	MW306190
Kabardino-Balkaria19/WB-964	Russia	2019	MT459800
Kaliningrad 18/WB-9766	Russia	2018	OM966718
Kaliningrad 19/WB-10168	Russia	2019	OM966719

Supplementary Table S2. Synonymous SNPs of seven ASFVs under study compared to Georgia 2007/1 (FR682468.2), Tanzania/Rukwa/17/1 (LR813622) and MAL/19/Karonga (MW856068) with nucleotide substitutions on amino acids highlighted in grey.

ORF	SNP/AA	Georgia 2007/1 (FR682468.2)	MOZ/01/2005	RSA/08/2019	ZIM/2015	MAL/04/2011	MAU01/2007	MAD/01/1998	TAN /01/2011	MAL/19/Karonga (MW856068)	Tanzania/Rukwa/17/1 (LR813622)
MGF360-3L	G 95 G	T	T	T	T	T	T	T	C	C	C
MGF360-4L	Y 306 Y	A	A	A	A	A	G	G	A	A	A
MGF360-9L	G 79 G	A	A	A	A	G	G	G	G	G	G
MGF360-10L	D 312 D	G	G	G	G	G	A	G	G	G	G
MGF360-14L	L 223 L	C	C	C	C	T	C	C	C	C	C
MGF505-6R	E 328 E	G	G	G	G	G	A	G	G	G	G
MGF505-6R	R 424 R	G	G	G	G	A	G	G	G	G	G
MGF505-7R	L 27 L	G	G	G	G	A	G	G	G	G	G
MGF360-15R	L 241 L	C	C	C	C	C	T	C	C	C	C
MGF360-15R	N 253 N	C	C	C	C	C	T	C	C	C	C
A179L	F 199 F	A	A	A	A	A	G	G	G	G	G
F334L	Q 147 Q	C	C	C	C	C	C	C	T	T	T
EP424R	G 10 G	G	G	G	G	G	G	G	A	A	A
EP152R	H 126 H	C	C	C	C	C	C	C	T	T	T
EP402R	I 118 I	C	T	T	T	T	T	T	T	T	T
M1249L	P 124 P	G	G	G	G	G	G	G	A	A	A
M1249L	A 1046 A	T	T	T	T	T	T	T	G	T	T
C717R	A 554 A	C	C	T	C	C	C	C	C	C	C

**C962R**	**K 345 K**	**A**	**A**	**A**	**G**	**A**	**A**	**A**	**A**	**A**	**A**
**B646L**	**L 477 L**	**G**	**G**	**A**	**G**	**G**	**G**	**G**	**G**	**G**	**G**
**B125R**	**V 39 V**	**G**	**G**	**A**	**G**	**G**	**G**	**G**	**G**	**G**	**G**
**G1340L**	**G 74 G**	**A**	**A**	**A**	**A**	**A**	**G**	**G**	**G**	**G**	**G**
**G1340L**	**S 910 S**	**G**	**G**	**G**	**G**	**G**	**G**	**G**	**C**	**C**	**C**
**G1340L**	**Q 1056 Q**	**C**	**C**	**C**	**C**	**T**	**C**	**C**	**C**	**C**	**C**
**O61R**	**I 18 I**	**A**	**A**	**A**	**C**	**C**	**C**	**C**	**C**	**C**	**C**
**NP868R**	**S 830 S**	**C**	**C**	**C**	**C**	**C**	**C**	**C**	**T**	**T**	**T**
**D339L**	**E 103 E**	**T**	**T**	**T**	**T**	**T**	**C**	**T**	**T**	**T**	**T**
**D1133L**	**L 554 L**	**A**	**A**	**A**	**A**	**A**	**G**	**G**	**G**	**G**	**G**
**D345L**	**D 31 D**	**G**	**G**	**G**	**G**	**G**	**G**	**G**	**A**	**A**	**A**
**E183L**	**R 170 R**	**T**	**T**	**T**	**T**	**T**	**T**	**T**	**C**	**T**	**T**
**E183L**	**Y 150 Y**	**G**	**G**	**G**	**G**	**G**	**G**	**G**	**A**	**G**	**G**
**E301R**	**G 10 G**	**C**	**C**	**C**	**C**	**T**	**C**	**C**	**C**	**C**	**C**
**E301R**	**P 12 P**	**G**	**G**	**G**	**A**	**G**	**G**	**G**	**A**	**A**	**A**
**EP296R**	**H 207 H**	**T**	**T**	**T**	**T**	**T**	**T**	**T**	**C**	**T**	**T**
**EP296R**	**V 209 V**	**G**	**G**	**G**	**G**	**G**	**G**	**G**	**A**	**A**	**A**
**I243l**	**I 136 I**	**G**	**G**	**G**	**G**	**A**	**G**	**G**	**G**	**G**	**G**
**I215L**	**P 2 P**	**T**	**T**	**C**	**T**	**T**	**T**	**T**	**T**	**T**	**T**
**MGF505-11L**	**R 384 R**	**A**	**A**	**G**	**A**	**A**	**A**	**A**	**A**	**A**	**A**
**MGF100-3L**	**K 20 K**	**C**	**C**	**C**	**T**	**C**	**C**	**C**	**C**	**C**	**C**
**L11L**	**L 6 L**	**G**	**G**	**G**	**G**	**G**	**G**	**G**	**A**	**A**	**A**

Supplementary Table S3. Non-synonymous SNPs identified between nine ASFV genotype IIs from Africa compared to Georgia 2007/1.

ORF	Position	Georgia 2007/1 (FR682468)	MOZ/01/2005	RSA/08/2019	ZIM/2015	MAL/04/2011	MAU/01/2007	MAD/01/1998	TAN /01/2011	MAL /19/ Karonga (MW856068)	Tanzania /Rukwa/17/1 (LR813622)
MGF360-3L	104	C	C	C	C	C	C	C	Y	Y	Y
MGF110-1L	109	W	W	W	W	W	W	W	R	R	R
MGF110-3L	91	G	G	G	D	G	G	G	G	G	G
MGF110-4L	101	D	D	D	E	D	D	D	D	D	D
MGF110-4L	17	V	V	V	V	V	L	L	V	V	V
MGF110-5L-6L	35	L	L	L	H	L	L	L	L	L	L
MGF110-5L-6L	164	S	S	S	S	S	S	S	-	-	-
MGF110-8L	1	V	V	V	V	V	V	V	M	M	M
MGF110-8L	81	T	T	T	T	T	T	T	M	M	M
MGF110-8L	94	G	G	G	G	G	G	G	I	I	I
MGF110-13LB	115	Q	Q	Q	Q	R	Q	Q	Q	Q	Q
MGF110-13LB	102	L	L	L	L	S	L	L	L	L	L
MGF360-6L	318	L	L	L	L	F	L	L	L	L	L
MGF360-12L	35	D	D	D	D	N	D	D	D	D	D
MGF360-13L	177	G	G	G	D	D	D	D	D	D	D
MGF360-14L	351	P	P	P	P	P	L	L	P	P	P
MGF505-4R	231	P	P	P	P	P	L	L	L	L	L
MGF505-4R	321	I	I	I	N	I	I	I	I	I	I

MGF505-7R	249	P	P	P	L	P	P	P	P	P	P
MGF505-9R	39	R	R	R	R	Q	R	R	R	R	R
MGF360-15R	178	A	A	A	A	A	V	A	A	A	A
A238L	41	C	C	C	C	C	C	C	Y	Y	Y
A238L	53	C	C	C	C	C	C	C	Y	Y	Y
A859L	427	A	A	A	E	E	E	E	E	E	E
F317L	230	R	R	R	R	R	R	R	H	H	H
F778L	447	A	A	A	A	A	A	A	V	V	V
EP402R	201	T	T	T	I	T	T	T	T	T	T
C257L	78	T	T	T	T	T	T	T	M	M	M
B169L	151	D	D	N	D	D	D	D	D	D	D
G1340L	808	S	S	S	N	S	S	S	S	S	S
G1211R	108	V	V	I	V	V	V	V	V	V	V
G1211R	1002	N	N	N	N	I	I	I	I	I	I
CP2475L	1566	S	S	G	S	S	S	S	S	S	S
D345L	288	M	M	M	M	M	M	M	T	T	T
H339R	68	M	M	I	M	M	M	M	M	M	M
H339R	121	S	S	N	S	S	S	S	S	S	S
QP509L	351	L	L	L	D	L	L	L	L	L	L
E183L	173	N	N	N	N	N	N	N	S	N	N
E199L	125	E	E	V	E	E	E	E	E	E	E
E199L	104	Q	Q	Q	H	Q	Q	Q	Q	Q	Q
E165R	130	D	D	D	D	D	D	D	N	N	N
I267L	129	I	I	I	I	I	V	V	V	V	V
I215L	173	A	A	V	A	A	A	A	A	A	A

**I215L**	**4**	**R**	**R**	**R**	**R**	**G**	**R**	**R**	**R**	**R**	**R**
**I196L**	**122**	**T**	**T**	**T**	**T**	**A**	**T**	**T**	**T**	**T**	**T**
**I196L**	**50**	**S**	**S**	**S**	**S**	**N**	**S**	**S**	**S**	**S**	**S**
**MGF505-11L**	**83**	**A**	**A**	**A**	**A**	**A**	**A**	**A**	**T**	**T**	**T**
**MGF505-11L**	**334**	**L**	**L**	**L**	**L**	**P**	**P**	**P**	**P**	**P**	**P**
**MGF100-3L**	**85**	**D**	**D**	**D**	**G**	**D**	**D**	**D**	**D**	**D**	**D**
**I10L**	**40**	**C**	**C**	**C**	**C**	**C**	**C**	**C**	**Y**	**Y**	**Y**
**MGF360-19Rb**	**42**	**I**	**I**	**I**	**I**	**T**	**I**	**I**	**I**	**I**	**I**

Supplementary Table S4. SNPs observed within the intergenic regions of the nine African Isolates and Georgia 2007/1 with differences highlighted in grey.

Position	IGR	Georgia 2007/1 (FR682468.2)	MOZ/01/2005	RSA/08/2019	ZIM/2015	MAL/04/2011	MAU/01/2007	MAD/01/1998	TAN /01/2011	MAL/19/Karonga (MW856068)	Tanzania/Rukwa/17/1 (LR813622)
5440	MGF110 4L / 5L	C	C	C	C	C	C	C	T	T	T
21151	MGF360-8L / 9L	A	A	A	A	A	A	A	-	-	-
24553	MGF360-11L / MGF505-1R	C	C	T	C	C	C	C	C	C	C
169470	I73R / I329L	-	-	-	-	-	G	-	-	-	-
169471	I73R / I329L	-	-	-	-	-	A	-	-	-	-
169472	I73R / I329L	-	-	-	-	-	A	-	-	-	-
169473	I73R / I329L	-	-	-	-	-	T	-	-	-	-
169474	I73R / I329L	-	-	-	-	-	A	-	-	-	-
169475	I73R / I329L	-	-	-	-	-	T	-	-	-	-
169476	I73R / I329L	-	-	-	-	-	A	-	-	-	-
169477	I73R / I329L	-	-	-	-	-	T	-	-	-	-
169478	I73R / I329L	-	-	-	-	-	A	-	-	-	-
169479	I73R / I329L	-	-	-	-	-	G	-	-	-	-
179683	I73R / I329L	-	-	-	-	-	A	-	-	-	-
179684	I73R / I329L	-	-	-	-	-	T	-	-	-	-
179704	I73R / I329L	G	G	G	G	G	G	G	A	A	A
179932	I329L / I 215L	-	-	-	-	-	-	-	A	A	A

**181961**	**DP96R / MGF360-19R**	**-**	**-**	**-**	**-**	**-**	**A**	**A**	**A**	**A**	**A**
